# Eavesdropping and contagious alarming in bird communities

**DOI:** 10.3758/s13420-025-00678-z

**Published:** 2025-06-02

**Authors:** Federica Rossetto, Gonçalo C. Cardoso, Paola Laiolo

**Affiliations:** 1 Biodiversity Research Institute (CSIC, Oviedo University, Principality of Asturias), Campus de Mieres, Edificio de Investigación – 5ª planta, C. Gonzalo Gutiérrez Quirós s/n, 33600 Mieres, Spain; 2 CIBIO, Centro de Investigação em Biodiversidade e Recursos Genéticos, InBIO Laboratório Associado, and BIOPOLIS Program in Genomics, Biodiversity and Land Planning, Campus de Vairão, Universidade do Porto, 4485-661 Vairão, Portugal; 3 Pyrenean Institute of Ecology (IPE-CSIC), Avda. Montañana 1005, Campus de Aula Dei, 50059 Saragossa, Spain

**Keywords:** Communication, Interspecific interactions, Passerines, Playback experiments, Vocalizations

## Abstract

Eavesdropping on heterospecific alarm calls can provide valuable information about predator presence and therefore yield survival benefits. However, if, how, and why individuals react to heterospecific alarms is poorly known. If heterospecific alarms trigger a response, individuals might either stop their own vocal activity (acoustic suppression), to avoid being detected, or start alarming (acoustic stimulation), to warn conspecifics or deter predators. Here, we performed playback experiments with the alarms of 14 common forest passerine species and tested whether heterospecifics changed their acoustic behavior after playbacks and whether this involved suppression or stimulation. Moreover, we tested whether bird behavior was influenced by species-specific attributes such as escape capability, the propensity of being predated by raptors and carnivorous mammals, and the acoustic properties of alarms affecting detectability. Birds uttered alarms more frequently after playbacks than after silent controls, gave the same alarms as they normally use in response to conspecifics, and did not suppress their acoustic activity after playbacks. These results indicate that birds perceive threat from heterospecific alarms and are contagiously stimulated to alarm, rather than inhibited. Species with morphological attributes that promote agility and suffering low predation rates were more acoustically stimulated by heterospecific alarms, irrespective of the acoustic properties of the playback or their own alarms, indicating that the propensity to join into an alarm calling bout is mediated by predation risk. This study provides evidence for contagious alarming across species, for potential costs of responding to these stimuli, and shows a facilitative role of signalers within communities.

**Supplementary Information:**

The online version contains supplementary material available at 10.3758/s13420-025-00678-z.

## Introduction

Social information can be crucial in the decision-making of many animal species—for example, under anti-predatory and resource exploitation contexts. Such information may be obtained through signals that evolved for communication purposes (Smith & Harper, 2003), or through inadvertent social cues of threat, habitat, or resource quality (public information; Lea et al., 2008). The same mechanisms may operate among individuals of different species, through interspecific communication or inadvertent cues (Magrath et al., 2015a; Seppänen et al., 2007). Eavesdropping refers to mechanisms through which communication that is not directed at oneself is used in decision-making. Eavesdropping on conspecific signals is widespread (Mennill et al., 2002), but it can also be used on heterospecific signals (e.g., mammals: Shriner, 1998; birds: Johnson et al., 2004), even among distantly related species (e.g., between reptiles and birds: Vitousek et al., 2007; between birds and primates: Martínez et al., 2022). Overall, eavesdropping can have important fitness benefits and be paramount for the life of many species (Magrath et al., 2015a). Yet the commonness of eavesdropping in ecological communities, the type of response triggered, and the differences among species remain unclear.

A large number of studies have shown that birds are able to use information extracted from the acoustic signals of other avian species. For example, owls gather information on breeding site quality from heterospecific calls (Parejo et al., 2012), nuthatches obtain information about the characteristics of predators from black-capped chickadee alarms (Templeton & Greene, 2007), and many passerines eavesdrop on the signals of their predators (Dutour et al., 2017; Jarčuška, 2023). Experimental studies on the eavesdropping of avian alarm calls have mainly focused on a particular intraspecific context (e.g., to gain information on predator presence; Kalb & Randler 2019) or on eavesdropping on the signals of predators (e.g., Tilgar & Moks, 2015), but whether the interchange of information through alarm calls is extended to large numbers of species—for instance, all species of a guild or a community—has not been studied. Among the limited published information, Magrath et al. (2009) explored the response to ‘hawk calls’ of three passerine species, and Randler and Vollmer (2013) and Dutour et al. (2016) studied responses to mobbing calls. While the benefits of interspecific information flow, and its links with species diversity and interactions have been investigated (Goodale & Magrath, 2024; Reichert et al., 2024), no study has so far considered all avian species within a community and examined a wide range of different alarm calls.

To assess the generality of interspecific alarming within communities and its determinants, we studied bird responses to the alarm calls of other community members. Avian communities at mid-high latitudes represent a good study system to examine acoustic eavesdropping among heterospecifics because it is common, especially outside the breeding season, that individuals of different species forage communally (mixed-species flocks; Morse, 1970) or rest together (interspecific roosts; Beauchamp, 1999). These aggregations might be useful to reduce the risk of predation (Sridhar et al., 2009)—for example, through a ‘many-eyes’ effect (increased probability of a predator being noticed; Pulliam, 1973) or through a dilution effect (reduced probability of being targeted by a predator; Wrona, 1991). It is likely that, under these conditions, information transfer and acoustic interactions among different species occur. Outside the breeding season, the most important acoustic signals employed by passerine birds are calls, of which ‘alarms’ are calls prompted by the presence of a threat (Catchpole & Slater, 2003). Alarms are used to warn conspecifics of danger or to recruit conspecifics or heterospecifics to harass predators (mobbing calls) or under immediate physical threat (distress calls; Bradbury & Vehrencamp 1998; Conover, 1994; Marler, 2004). There are also functionally referential alarm calls, varying with predator size, the level of perceived threat, and the hunting behavior of the predator (Griesser 2008; Soard & Ritchison, 2009; Templeton et al., 2005).

When eavesdropping on heterospecific alarms, birds might start alarming themselves as an anti-predatory strategy (to warn conspecifics or deter predators) or reduce their vocal activity, as a strategy to avoid being detected by predators. The former behavior is referred to as ‘Acoustic stimulation’ (Magrath et al., 2015a; Randler & Vollmer, 2013) and the latter ‘Acoustic suppression’, which is the acoustic component of the ‘freezing response’ (interruption of any activity; Haftorn, 2000; Hetrick & Sieving, 2012). We tested whether acoustic stimulation or suppression occurred (acoustic stimulation vs. acoustic suppression hypotheses) by conducting playback experiments. Specifically, we broadcast several types of alarm calls of different species to determine whether birds eavesdropped on heterospecific alarm calls. We also assessed whether they were stimulated to alarm themselves or whether this inhibited them from doing so. Furthermore, we analyzed the acoustic characteristics of the alarms uttered after stimulation. We compared the responses to heterospecific playbacks with the alarms given after conspecific playbacks to see if there were any differences in the acoustic features of the responses. Finally, we examined the intrinsic drivers of species differences in response to heterospecific alarms. We tested whether species-level features—such as vulnerability to predation, morphological features indicating escape capability, and acoustic features affecting alarm detectability—influenced the behavior of eavesdropping birds. Next, we tested whether the responses varied depending on the type of alarm broadcast, and whether familiarity between species influenced the response in terms of previous experience with heterospecific vocalizations (Magrath et al., 2015b). We also tested whether the response was stronger between species with a similar ecology, suggesting the potential for competitive interactions (Laiolo, 2012; Reed, 1982). If eavesdropping occurs, we predict that playback experiments would provoke a behavioral response. Considering the trade-off between the risks and benefits of alarming, the interaction could be either negative (birds become silent after the stimuli), supporting the acoustic suppression hypothesis, or positive (birds start alarming after the stimuli) supporting the acoustic stimulation hypothesis. Regarding species level traits, species with alarms that are more easily detectable by predators (Fallow & Magrath 2010; Forsman & Mönkkönen 2001; Leavesley & Magrath, 2005) should be less stimulated to vocalize or should suppress their vocal activity. By contrast, species that become more stimulated should be the ones that take less risk when alarming (i.e., less vulnerable, more agile; Leavesley & Magrath, 2005), but also the opposite could be expected when the threat is strong (those that have the higher probability of being predated; Møller et al., 2006). Lastly, since higher familiarity among species may help recognize interspecific alarms (Magrath et al., 2015b) or lead to habituation (Jaimipak et al., 2019), interactions (either stimulation or suppression) could also vary with the degree of species co-occurrence. While testing these hypotheses, we found that a large number of coexisting species responds to the alarm calls of other species and apparently modulate their responsiveness depending on their vulnerability to predators.

## Methods

### Playback experiments

In 2020, 2021, and 2022, we performed a total of 122 playback trials of alarm calls to test for heterospecific interactions. Trials were conducted in autumn (from September to November), to minimize the effect of sexual interactions, in 84 localities separated from each other by more than 400 m. This distance was selected to avoid testing for the same individuals in consecutive tests along the same transects, to avoid overlap in individual home range size (Johnstone, 1998; Rolando, 1998). Trials were performed in good weather conditions from 7:30 a.m. to 1:00 p.m., which is the time period with the highest bird activity in autumn and winter (Laiolo et al., 2003). The study area covered 58,270 hectares (Fig S1) and consisted of deciduous forests, mainly composed of *Castanea sativa* interspersed with *Quercus robur* and *Fagus sylvatica* in Asturias, Northern Spain. We broadcast alarms of 14 species in the local community, one species per trial. The 14 species for which we broadcasted alarm calls were: *Certhia brachydactyla, Cyanistes caeruleus, Erithacus rubecula, Fringilla coelebs, Lophophanes cristatus, Parus major, Periparus ater, Poecile palustris, Regulus ignicapilla, Sitta europaea, Sylvia atricapilla, Troglodytes troglodytes, Turdus merula,* and* Turdus philomelos.*

In all 14 species, alarm calls were clearly differentiated from songs and other calls (see references in Table S1 and Cramp et al., 2004) and elicited different kinds of responses in the local community (Rossetto & Laiolo, 2024). The broadcast stimuli were obtained from recordings downloaded from the xeno-canto repository (https://xeno-canto.org/) by selecting alarm recordings with low background noise that were of high quality (when possible, graded as quality ‘A’ in xeno-canto) and recorded close to our study area (see Rossetto & Laiolo, 2024, for the reliability of this source of tracks). After selecting and downloading the recordings, tracks were prepared using Avisoft-SASLab Pro (Specht, 2002). We classified the downloaded calls as either ‘narrow-sense’ alarms or ‘broad-sense’ alarms. Narrow-sense alarms refer to the sounds most commonly used by each species known to warn of predator threats (sensu Cramp et al., 2004). Since the species considered in this study emit both territorial calls and alarm calls, we ensured that the type of calls selected and downloaded from the xeno-canto repository to be broadcast were recorded in previous studies in the presence of predators, to have a strong support of their antipredator function (see references in Table S2). Broad-sense alarms include the most commonly used alarms (narrow-sense alarms) plus other types of alarms uttered less frequently, but always given in an antipredator context (Cramp et al., 2004, and references therein; Suzuki, 2020). Half of the tracks broadcast included broad-sense alarms, and the other half was composed of narrow-sense alarms. A total of 28 tracks with alarms (of 46 individuals) were broadcast.

Playback stimuli were broadcast with ‘Vieta pro easy’ omnidirectional loudspeakers (80 Hz- 20 kHz response) positioned at less than one meter above the ground. The study woodlands are generally steep and thus even if positioned close to the ground, the loudspeaker could be at the same height of birds located in a tree downhill. Each playback trial lasted 8 minutes: 4 minutes of silence followed by 2 minutes in which alarm calls of one species were broadcast and then 2 minutes of silence again. Additionally, we performed 36 trials in which no sound was broadcast (silent controls), therefore consisting of 8 minutes of silence. For each playback species, we conducted from 7 to 12 trials (Table S1). During the trials, two observers with experience in aural bird censuses were positioned 20 m from the loudspeaker, in opposite directions, and noted the species alarming in a radius of 20 meters around the loudspeaker. We refer to these responding species as “focal species”, as opposed to the “playback species” whose stimuli were broadcast. The focal species consisted of: *Aegithalos caudatus, Certhia brachydactyla, Cyanistes caeruleus, Erithacus rubecula, Fringilla coelebs, Garrulus glandarius, Lophophanes cristatus, Parus major, Periparus ater, Phylloscopus collybita, Poecile palustris, Pyrrhula pyrrhula, Regulus ignicapilla, Sitta europaea, Sylvia atricapilla, Troglodytes troglodytes, Turdus merula.* Additionally, one AudioMoth recorder (Version 1.1.0; Hill et al., 2018) was placed near the loudspeaker (at a distance of approximately 40 cm) in order to confirm the identification of the focal species in case it could not be identified in the field. We compared uncertain vocalizations with recordings and sonograms downloaded from the online xeno-canto repository (https://xeno-canto.org/) for the species pool of the study area (Laiolo et al., 2018).

After identification, we classified trials on the basis of the behavior observed to test whether it supported the ‘acoustic stimulation hypothesis’ or the ‘acoustic suppression hypothesis’. We considered two types of response to the playback: acoustic suppression and acoustic stimulation. Following the procedure described in Rossetto and Laiolo (2024), if a species alarmed during the 4 min before playback stimulus (or during the first 4 min in the case of silent controls) and stopped for the 4 min after the playback stimulus, it was considered a ‘suppression’ event. Similarly, if a species did not alarm during the 4 min before the playback and alarmed during the 4 min after the playback stimulus (or during the last 4 min of silence in the case of silent controls) it was considered a ‘stimulation’ event. Our assignment of acoustic stimulation is conservative because it does not include instances when a species was already alarming during the 4 minutes before the playback. In order to quantify the acoustic stimulation or suppression responses of each focal (responding) species to each playback species, we calculated the percentage of stimulation events (or, in separate analysis, suppression events) observed in response to each playback species. Here, suppression events and stimulation events refer to trails in which a response (suppression or stimulation, respectively) was recorded. The percentage was calculated as the number of such events divided by the number of trials conducted with the playback species. For each focal species, the responses to playback of conspecifics were not considered. Then, for each focal species, we calculated the average of all the percentages of suppression events and the average of all the percentages of stimulation events representing the overall frequency of suppression and overall frequency of stimulation after heterospecific playbacks, respectively. Additionally, for each focal species, we used the same procedure to compute the percentage of suppression and stimulation to silent controls. We measured the peak sound pressure level (SPL) of tracks with a Realistic Sound Level Meter 33–2055 (C-weighting, ‘fast’ response) at 1-m distance and maintained it in the range 71–90 dB (peak 82.14 dB, Table S1), for them to be clearly distinguishable at a distance of 20 m (our range of observation) but respecting the natural amplitude variation due to body size differences among species (Podos & Patek, 2015). Moreover, the location of focal birds, and thus their distance from the sound source and in turn the perceived intensity, could not be controlled a priori in these community-wide experiments. In any case, the peak SPL of playbacks had no discernible effect on the behavior of birds since it was unrelated to both suppression and stimulation events (Table S3).

For each focal species, we also compared the spectro-temporal characteristics of sounds emitted after playback of heterospecific and conspecific alarm calls. We identified and classified the alarms that were most frequently employed in response to heterospecific and to conspecific alarm playbacks, which were also all alarm calls. For each focal species, three alarms were sampled from recordings of the heterospecific experiments for up to three individuals, when possible, and the same procedure was repeated for conspecific stimuli. We selected clear isolated alarm calls in tracks with high signal to noise ratio. We measured the following variables: peak frequency (in log_10_Hz), frequency bandwidth (the difference between maximum and minimum frequencies, both in log_10_Hz) and duration (log_10_s). Measurements were made using Avisoft SASLab Pro (sampling rate = 22050 Hz; FFT length = 512; window = ‘hamming’; frequency resolution = 43 Hz; intensity = 0). Alarm duration was measured on the oscillogram, peak frequency (the frequency with the highest cumulative amplitude), minimum and maximum frequency were measured on the power spectrum, and frequency bandwidth was measured as the difference between maximum and minimum frequencies (Fig. S2). Sound frequency was taken on a ratio scale (logarithm of Hz) to prevent overestimating bandwidth or frequency differences in species using alarms with higher sound frequencies relative to those using lower frequencies (Cardoso, 2013). In addition, duration measurements were also taken on a ratio scale (logarithm of s) because duration and other quantitative properties of stimuli are generally perceived as ratios (Akre & Johnsen, 2014).

### Species-specific features favoring responses to heterospecific alarm calls

We considered a set of variables—both for the species used in the playback recordings and for each focal species responding to playbacks—that could influence alarm behavior in response to heterospecific alarms. To control for sampling effects and random responses, the abundance of each species in the area was considered (more abundant species have the opportunity to be heard more). Abundance was estimated through several surveys conducted from 2010 to 2022 (Laiolo et al., 2018), but given that it changed little over the years, for this study we considered the abundance in 133 plots surveyed in autumn 2022 (the closest survey in terms of time, carried out during the study), performed prior to playbacks or on different days. Surveys were carried out by walking and stopping every 400 m to record all visual and aural detections in a 100-m radius around the observer during a 10-min period. Following Laiolo et al. (2018), observers remained still in the plot center for 5 min and walked for the remaining 5 min to flush out possible hidden individuals. As intrinsic features, we estimated an index of predation rate that took into account the main avian and carnivore predators. For this, we considered indices of predation by raptors and by domestic cats *Felis catus*, which are the most common predators of passerines in the area (personal observation). To account for predation rate by raptors, we used the metrics of prey vulnerability developed by Møller and Nielsen (2007), corresponding to the observed log_10_-transformed number of prey minus the log_10_-transformed expected number of prey of *Accipiter nisus* and *A. gentilis*. These raptors specialize on passerines and are both frequent in the study area (personal observation) and across much of Europe. For each species, we averaged the predation rate indices by Møller and Nielsen (2007) for *A. nisus* and *A. gentilis*. Since two species (*Regulus ignicapilla* and *Certhia brachydactyla*) were not present in Møller and Nielsen’s (2007) database, we used the predation indices of the phylogenetically closest species (*Regulus regulus*, *Certhia familiaris*). For three species (*Periparus ater*, *Regulus regulus* and *Troglodytes troglodytes*), no predation event by *A. gentilis* was recorded, and therefore we set these missing values to the lowest predation rate available in the database. The predation rates estimated by Møller and Nielsen (2007) showed consistency in vulnerability across large spatial scales (Møller & Ibáñez-Álamo, 2012) and with raw predation data from the literature (Table S4). The mean predation rate we obtained from the literature survey (Table S4) was correlated with the predation rate estimated by Møller and Nielsen (2007) for our sample of species (*r* =.64, *N* = 16 species, *p* <.01). To estimate predation from cats, we used the data published in Woods et al. (2003) on the percentage of prey brought home by domestic cats. An index of vulnerability to cat predation was also reported by Møller and Ibáñez-Álamo (2012), but not for all of our focal species. It was nonetheless correlated (*N* = 5 species; *r* =.7) with that of Woods et al. (2003), which we used here.

We also estimated a proxy of the escape capability for each species, considering its body mass, flight maneuverability, and foraging height in vegetation. Smaller birds and birds with higher flight maneuverability should be more agile when fleeing from predators (Lima, 1986; Tellería et al., 2001). Also, species foraging in the tree canopies should be more protected from potential predator attacks and be more difficult to detect (Ekman, 1986; Suhonen, 1993). We obtained body mass data for each species from the literature (Laiolo et al., 2015) and log-transformed them to reduce skewness in the data. As a proxy of flight maneuverability, we obtained hand-wing index (HWI, a morphological metric linked to wing aspect ratio) for each species from Sheard et al. (2020). More elliptical wings (i.e., lower HWI) should provide more efficient maneuverability (Ocampo et al., 2023). Finally, we assigned to each species a value associated with its predominant tree height position during foraging outside the breeding season, following Carrascal et al. (1990) and Laiolo et al. (2003). We assigned a value of 0 to species mostly foraging on the ground, 0.5 to species foraging on tree trunks and low branches, and 1 to species mostly foraging in canopy foliage. To summarize the variables of escape capability (i.e., body size, HWI, and tree height position) and reduce their covariation, we performed a principal component analysis (PCA) on scaled variables (R package *stats*). We retained the first component (hereafter PC1-escape) from the PCA, which was related positively to the vertical position and negatively to the body size of each species (Table 1); the second PC explained less variance and had weak correlations with all variables.

**Table 1:** Results of principal component analyses carried out on species morphological and ecological characteristics associated with escape capability (left), and on acoustic variables associated with detectability (right)

Escape capability		Acoustic detectability	
	PC1-escape		PC1-sounds
Hang-wing index HWI	0.201	Frequency peak	0.463
Tree height position	0722	Bandwidth	−0.658
Body mass	−0.662	Duration	−0.595
Eigenvalue	1.129	Eigenvalue	1.318
Total variance (%)	42.5	Total variance (%)	57.9

Since species that are more stimulated by heterospecifics should have alarms that are less acoustically detectable or localized by predators (i.e., shorter, higher-pitched and/or with low frequency bandwidth; Klump, 2000), we estimated the degree of ‘acoustic detectability’ of alarm calls by their acoustic features: frequency peak (log_10_Hz), frequency bandwidth (log_10_Hz), and duration (log_10_s). We used the acoustic measurements of alarms after heterospecific stimuli only, as we were interested in the response to heterospecific alarms. For each species, we calculated the average peak frequency, frequency bandwidth, and duration, and, because these traits covary to some extent, we summarized them into one axis using PCA. We retained PC1 (hereafter PC1-sound), which was positively correlated with peak frequency and negatively correlated with bandwidth and duration (Table 1). High values of this PC1 thus indicate alarms that are more difficult to detect or localize (Klump, 2000). To test whether acoustic characteristics of the sounds broadcast determine the response elicited, we repeated the same procedure to estimate the PC1 of acoustic parameters of the type of alarm most frequently broadcast for each playback species.

Because behavioral responses might be determined by daily interactions and previous encounters between species, we estimated an index of co-occurrence between playback species and responding species as a proxy of their degree of familiarity. To estimate co-occurrence, we built a presence/absence matrix using data from the aforementioned survey (each plot was a row and each target species a column). The *C-score* (Stone & Roberts, 1990) was then calculated from this presence/absence matrix to quantify the propensity of species to co-occur, as:

1 $${C}_{ij}=\left({R}_{i}-D\right)\times \frac{\left({R}_{j} -D\right)}{{R}_{i}\times {R}_{J}}$$

where ${C}_{ij}$ is the *C-score* for species *i* and *j*, ${R}_{i}$ and ${R}_{J}$ are the number of occurrences of species *i* and *j*, respectively, and $D$ is the number of plots where species *i* and *j* co-occur. This index was scaled to range from zero (indicating sympatry) to 1 (indicating allopatry; Gotelli & Rohde, 2002; Laiolo et al. 2017). C*-scores* were calculated with the R package *bipartite* (Dormann et al., 2008).

Finally, to ensure that the observed response was not due to territorial behavior and to further confirm that the alarms broadcast had an anti-predatory rather than territorial function, prior to all analyses we tested whether stimulation events were more common in closely related species or that share their ecological requirements, as these species are more likely to compete and defend heterospecific territories through calls (Laiolo, 2012; Reed, 1982). Phylogenetic distance and trophic niche dissimilarities among species were estimated as indicated in Tables S5–S6.

### Data analyses

#### Behavioral response

To test whether species were suppressing their vocal activity or were stimulated by the playback of heterospecific alarms, we compared the suppression and the stimulation percentages among treatments (playback vs. silent controls) to test the ‘acoustic suppression hypothesis’ and the ‘acoustic stimulation hypothesis’, respectively. We tested for normality for the differences between pairs of percentages of events by means of the Shapiro–Wilk test, and then used a paired *t* test in cases where the distribution did not differ significantly from normality, or the Wilcoxon signed-ranks test in cases where the distribution was significantly non-normal. We also repeated these analyses using data from playbacks of narrow- or broad-sense alarms, separately.

We fitted generalized linear mixed models (GLMMs) with the *glmmTMB* package (Brooks et al., 2017) to test whether the heterospecific response to alarm playbacks were correlated with trophic or/and phylogenetic distance between species, as expected in case of competitive interactions among species through calls. The binary variable of stimulation/no stimulation was set as response variable, the playback species and experimental plot as random factors and the phylogenetic distance/trophic dissimilarity between playback and focal species as the fixed term. We ran two separate tests, as phylogenetic and niche distances were highly correlated, with a binomial error and logit link function.

#### Acoustic response and type of alarm call employed

To investigate which vocalizations were used in response to playbacks, we analyzed the recorded alarms of the ten most common species (*Cyanistes caeruleus*, *Erithacus rubecula*, *Fringilla coelebs*, *Garrulus glandarius*, *Parus major*, *Periparus ater*, *Regulus ignicapilla*, *Sitta europaea*, *Troglodytes troglodytes*, *Turdus merula*, all present in >30% of survey plots). First, we identified each species’ alarm from recordings and qualitatively assessed whether the same type of alarm was the most common in heterospecific and conspecific contexts. We then quantified differences among the most common alarm in the two contexts by running GLMMs (Baayen, 2008) with the *glmmTMB* package, fitting variation of frequency peak, frequency bandwidth, or alarm duration as the dependent variable. As a fixed effect, we considered the type of context, a variable with two levels indicating whether the alarm was uttered after conspecific or heterospecific playbacks, and, as a random factor, the identity of the focal species.

#### Difference in responses among species

We designed GLMMs to investigate differences between species in responsiveness to playback. The response variable in these models was a binary variable indicating whether each focal species was stimulated or not by the playback in a given trial or, instead, whether each focal species suppressed or not its vocal activity with the playback in a given trial. From this point forward, we ran the models only for stimulation responses, and not for suppression responses, as we found no consistent suppression responses in any of the species (see Results), and we thus refer to stimulation for simplicity. These models had a binomial error structure and logit link function, and we did not use data from playbacks of conspecific alarms or from silent controls without playback. Each model included the focal (responding) species (limited to the ten most common focal species; see list above) as a fixed term, and playback species and experimental plot as random factors.

To test whether the playback alarm of some species elicited more responses in heterospecifics than others, we ran another GLMM by setting the response to playback (as above, fitted with binomial error structure and logit link function) as the dependent variable, the playback species as a fixed term, and the responding species and experimental plot as random factors. The statistical significance of fixed effects in these models was evaluated using Type III analysis of variance (ANOVA; *car* package; Fox & Weisberg, 2018).

#### Determinants of responsiveness

To examine which species traits determine the degree of response to heterospecific alarm call playbacks, we performed a GLMM with the response to playback as a binary dependent variable indicating whether each focal species was stimulated or not by the playback in a given trial, and setting as fixed effects the proxy of escape capability (PC1-escape), the proxy of detectability (PC1-sound), predation rate by raptors, predation rate by domestic cats, and degree of co-occurrence with the playback species (*C-score*). The log-transformed abundance of each species was also included to account for sampling effects. Random factors were the responding species, the playback species, and the experimental plot. We used a logit link and a binomial distribution for the error term. Collinearity among the predictors was inspected with a variance inflation factor (VIF) using the R package *performance* (Lüdecke et al., 2021), and was not found to be an issue in our model (VIF < 2.4 for all predictors, threshold value = 3; Zuur et al., 2010).

Similarly, to test which traits of the playback species elicited greater response, we performed a GLMM, with response to playback as the dependent variable (binary variable: 0 = no stimulation elicited or 1 = stimulation occurred), the acoustic characteristics of the sound broadcast (PC1), and the type of alarm broadcast (to test whether the response depended on whether the stimulus broadcast was a narrow-sense or broad-sense alarm) as a fixed effect. We included as random factors the responding species, the playback species, and the experimental plot and used a logit link and a binomial distribution for the error term. We only tested acoustic characteristics of the playback species, as they are the only features that the listener could perceive from acoustic stimuli and could therefore determine the response (no decoy was placed in the trials).

We did not take into account phylogenetic signals, as we found no correlation between the propensity of stimulation and phylogenetic relatedness (see Results). All analyses were performed in R (Version.3.6.1; R Core Team, 2013).

## Results

### Behavioral and acoustic responses, and differences among species

**Fig. 1:**
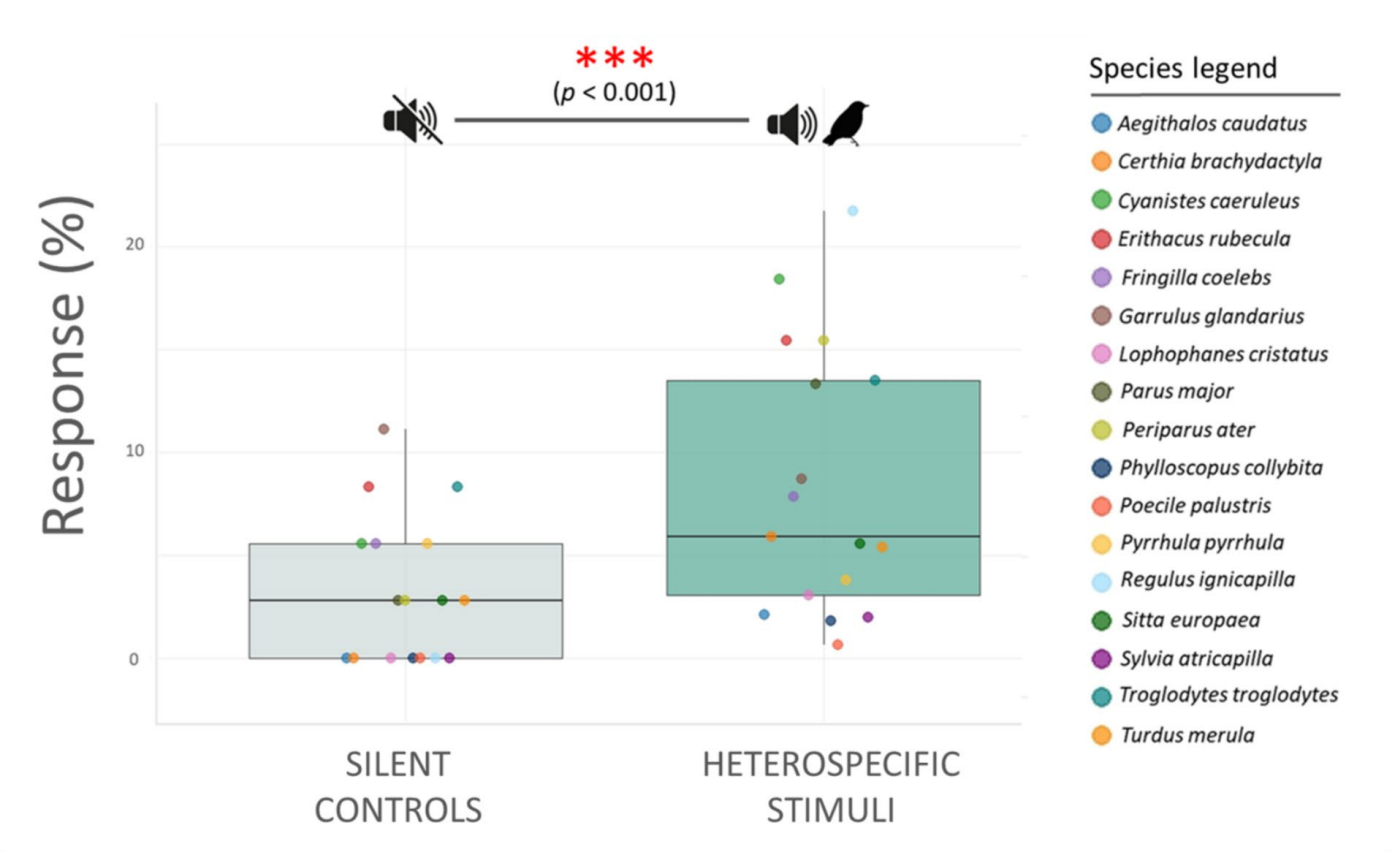
Boxplot showing the differential responses of species to silence and heterospecific alarm call playbacks (including both broad and narrow sense alarms). Box plots show medians, interquartile range (IQR), and extent of data to ±1.5 × IQR. Each dot is a species. The black line and red asterisk indicate significant differences as tested by means of a Wilcoxon signed-ranks test. (Color figure online)

Species acoustic stimulation and alarming occurred more frequently after heterospecific stimuli than after silent controls (Shapiro–Wilk: *p* =.03; *N* = 17 species, Wilcoxon signed-ranks test: *p* <.01), suggesting that the alarm calls broadcast generally elicited a response in heterospecifics supporting the ‘acoustic stimulation hypothesis’ (Fig. 1). The percentage of acoustic suppression was not significantly different between playbacks and silent controls (Shapiro–Wilk: *p* =.40; *N* = 17 species, paired *t* test: *t*_16_ = 1.78, *p* =.09), thus suggesting that the ‘acoustic suppression hypothesis’ was as equally probable as the null hypothesis. Results were identical when repeating analyses only for trials in which narrow-sense alarms were broadcast (acoustic stimulation: Shapiro–Wilk: *p* <.01; *N* = 17 species, Wilcoxon signed-ranks test: *p* <.01; acoustic suppression: Shapiro–Wilk: *p* =.94; *N* = 17 species, paired *t* test: *t*_16_ = 1.82, *p* =.10), or only for trials in which broad-sense alarms were broadcast (acoustic stimulation: Shapiro–Wilk: *p* =.20; *N* = 17 species, paired *t* test: *t*_16_ = 2.27, *p* =.037; acoustic suppression: Shapiro–Wilk: *p* =.50; *N* = 17 species, paired *t* test: *t*_16_ = 1.76, *p* =.14). There was no evidence, however, that species start alarming after the alarms of closely related species or species with similar trophic niche, and the proxy of interspecific competition (GLMM phylogenetic distance: β_st_= 0.0004, *p* =.907; trophic niche dissimilarity: β_st_= 0.008, *p* =.948).

**Fig. 2:**
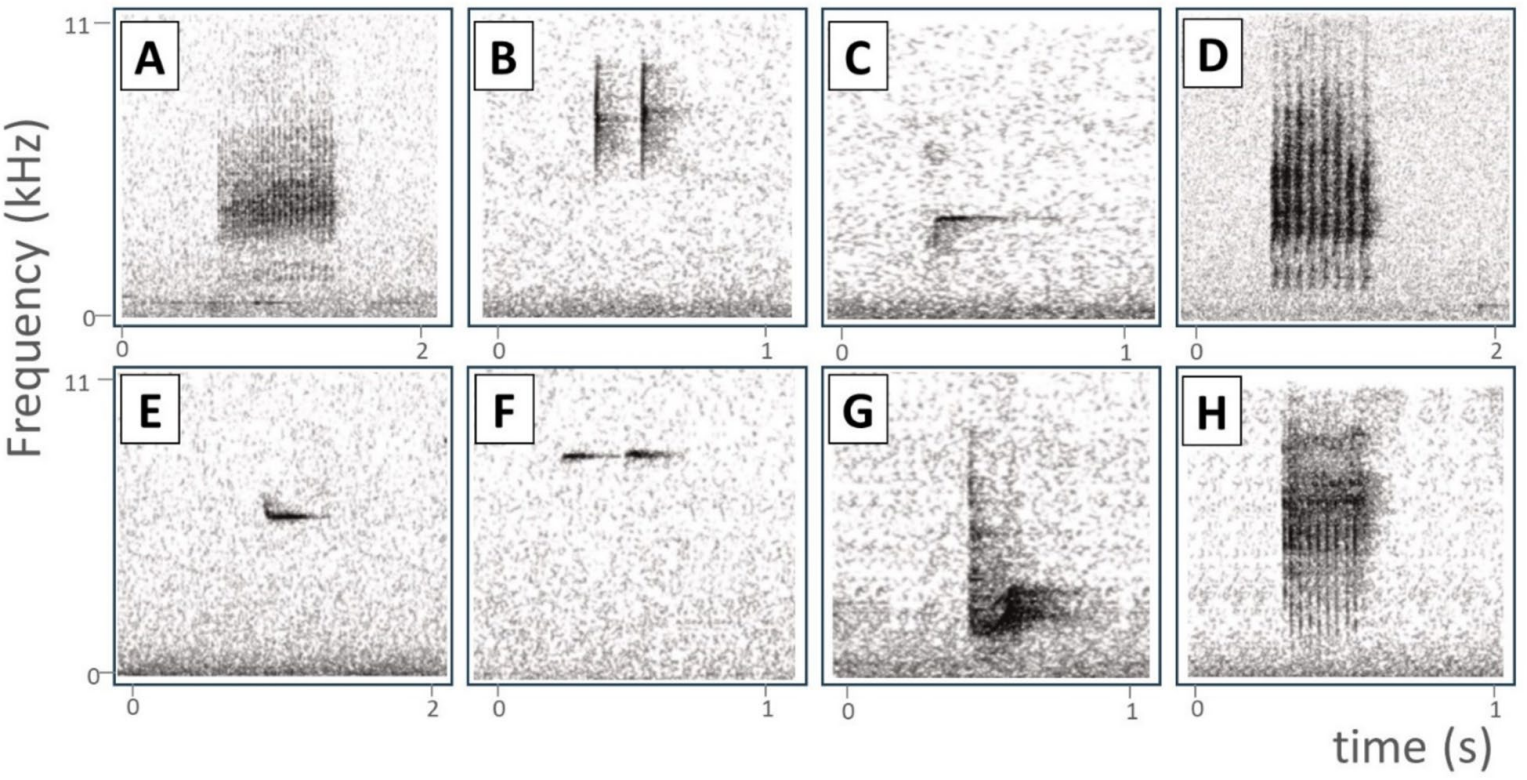
Spectrograms of the type of alarm most commonly employed by birds when stimulated by heterospecific and conspecific alarms (the same type is uttered in the two contexts). **A**: *Cyanistes caeruleus*; **B**: *Erithacus rubecula*; **C**: *Fringilla coelebs*; **D**: *Parus major*; **E**: *Periparus ater*; **F**: *Regulus ignicapilla*; **G**: *Sitta europaea*; **H**: *Troglodytes troglodytes*

We found that the category of the alarm most commonly employed when stimulated by heterospecific alarms corresponded with the one mostly employed with conspecifics (Fig. 2). Furthermore, we found no significant differences in peak frequency (*z* = −0.81; *p* =.42), frequency bandwidth (*z* = −1.32; *p* =.19), or duration of alarms (*z* = 1.25; *p* =.21) between the most commonly employed alarms after heterospecific stimuli and those employed after conspecific stimuli (total number of alarms analyzed = 94; Table S7). Moreover, the type of alarm most commonly uttered after heterospecific and conspecifics alarms was the ‘narrow-sense’ alarm for almost all species taken into account (except from *Erithacus rubecula* and *Periparus ater*; Fig. 2).

Some species responded more than other species to heterospecific alarms (ANOVA: χ^2^ = 28.547; *p* <.001). Even if the percentage of stimulation events was generally weak (8.51% on average), acoustic stimulation was particularly high in *Regulus ignicapilla* (21.75%), *Cyanistes caeruleus* (18.41%), and *Erithacus rubecula* (15.45%; Table S8, Fig. S3). On the other hand, no playback species elicited more responses than did others (ANOVA: χ^2^ = 16.112; *p* =.243).

### Determinants of responsiveness

**Fig. 3:**
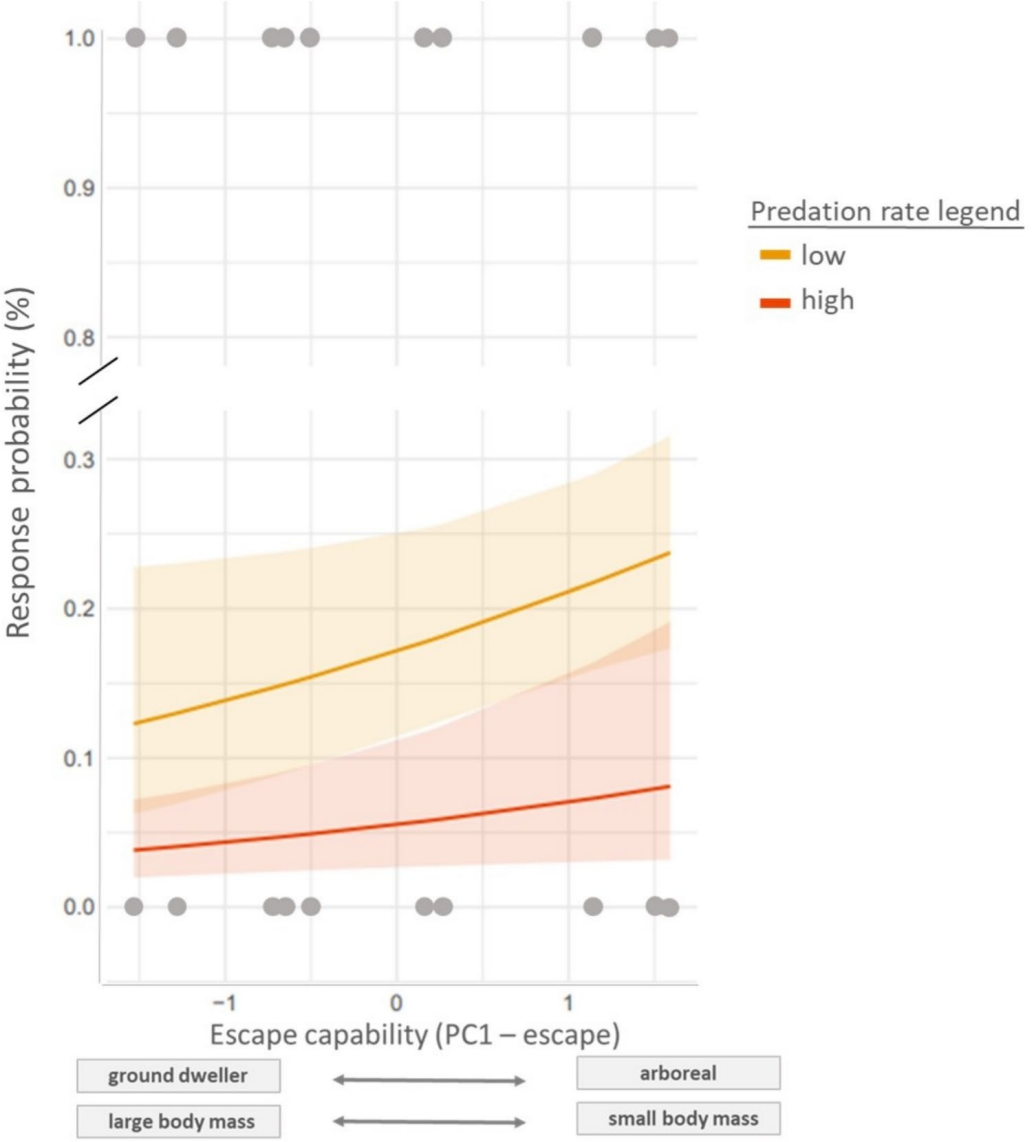
Relationship between the predicted probability of response (acoustic stimulation) to heterospecific alarm calls and species escape capability. The latter was expressed in terms of the first principal component of morphological and ecological variables proxies of escape capability, with the factors with the highest loadings indicated on the *x*-axis. The probability of response is represented controlling for the effects of acoustic detectability, predation rate by cats, co-occurrence with playback species (*C-score*) species abundance and predation rate by raptors. High levels of predicted values of predation rate by raptors (red line) correspond to a value of 2 while low levels of predicted values of predation rate by raptors (orange line) correspond to a value of −2. Each dot represents the response of focal species in a trial and the regression trend line and its confidence intervals are also shown. (Color figure online)

We found a significant and negative association between predation rate by raptors and the probability of a species of being stimulated by playbacks (β_st_ = −0.317, *p* =.027; Fig. 3a). We also found a significant and positive association between escape capability, as indicated by PC1-escape, and the probability of a species responding to heterospecific alarm playbacks (β_st_ = 0.257, *p* =.031; Fig 3b; full model results in Table 2). Overall, these results indicate that the species responding more are the ones that are less susceptible to predation by raptors and the ones more agile to escape. The probability of responding to the playback of heterospecific alarms was not predicted by any acoustic variable associated with the playback species, but heterospecifics were stimulated more by tracks of narrow-sense alarms than by tracks including multiple types of alarms (broad-sense alarms; Table 2).

**Table 2:** (a) Traits of responding species that predict their responsiveness (stimulation) and (b) traits of playback species that elicited a higher response

(a) Traits of responding species				
	*Estimate*	*SE*	*z*	*p*
Intercept	−5.355	1.770	−3.026	.002
PC1-escape	0.255	0.119	2.150	.032
PC1-sounds	−0.162	0.119	−1.360	.174
Predation rate by raptors	−0.315	0.143	−2.202	.028
Predation rate by cats	−0.058	0.114	−0.507	.612
Familiarity (co-occurrence; C-score)	−0.661	0.667	−0.991	.322
Species abundance	1.594	0.820	1.945	.052
(b) Traits of playback species				
	*Estimate*	*SE*	*z*	*p*
Intercept	−2.318	0.222	−10.459	<.001
PC1-playback sounds	−0.038	0.102	−0.373	.709
Type of stimulus (narrow-sense)	0.516	0.200	2.577	.010

*Note.* Generalized linear mixed models were run with 1,143 samples, controlling for species identity and study site as random factors. The significant predictors (at *p* <.05) are depicted in bold

## Discussion

Our playback experiments showed that birds eavesdrop on the heterospecific alarm calls of community members and are stimulated to alarm call themselves as they would in response to conspecifics. These responses to heterospecific alarms are likely evidence of an anti-predatory strategy based on cues that are neither provided by predators nor by conspecifics, but rather by signals of other community members (Gill & Bierema, 2013; Randler & Vollmer, 2013). This suggests that individuals decode signals of other species, even though they are used mostly in an intraspecific context. Therefore, there is feedback between intraspecific communication (the species originally alarming), interspecific eavesdropping, and again intraspecific communication (the eavesdropping species that then starts alarming).

### Generalized eavesdropping and acoustic stimulation

We found that birds were acoustically stimulated rather than inhibited by heterospecific alarm calls, supporting the hypothesis of acoustic stimulation, as opposed to the hypothesis of suppression that has been supported in some other studies, albeit with respect to different response behaviors (Haftorn, 2000; Hetrick & Sieving, 2012). Studies have found that birds ‘freeze’ after hearing alarm calls (i.e., become immobile to pass unnoticed; Sullivan, 1984), and this behavior also involve acoustic suppression. Since birds responded by alarming as they typically do in a conspecific context in our study, the behavior we observed likely functions to warn conspecifics of danger. This is also supported by the fact that the alarms uttered after heterospecifics matched with the category of ‘narrow-sense’ alarms, which are the alarms uttered in the presence of a predator (Cramp et al., 2004, and see references in Table S2), with the exception of *Erithacus rubecula* and *Periparus ater*, which might have a more heterogeneous alarm repertoire which should be further investigated. Apart from the support of literature on the antipredator significance of these calls, the observed stimulation did not involve species that are more likely to compete for the same resources (sharing evolutionary history or niche). Thus, these results do not support other potential interpretations of the observed stimulation, such as competition between playback and focal species, or at least suggests that anti-predatory behavior may prevail over competition. Future studies should be directed toward acquiring a larger sample of trials and increase the power of tests with different alarm types and thus elucidate the nuances in the behavior we observed.

We found no evidence that the playbacks of some species elicited more responses than the playbacks of others, indicating a generalized phenomenon of eavesdropping on the alarms of heterospecific community members, rather than on the alarms of only certain species or on specific types of alarms (e.g., mobbing, whose function is also to recruit other individuals). However, the narrow-sense alarms were the alarms eliciting higher stimulation, likely because these signals indicate the visual presence of a predator while other alarms calls are involved in other types of antipredator behavior. Nevertheless, although to a lesser extent, stimulation was still higher after broad-sense alarm calls compared to silent controls, indicating that those tests were nonetheless valid.

### Propensity of response linked to predation risk

We found differences among species in their propensity to respond to heterospecific alarm calls. Obtaining information on individual fitness in community-wide studies is generally unfeasible, but comparing the traits of more and less responsive species may indicate which costs or benefits shape their behavior (Salguero-Gómez et al., 2018). Our findings suggest that more agile species or species less susceptible to predation are more likely to respond. Namely, we observed a greater prevalence of acoustic stimulation in species that are either less preyed upon by raptors in the study area, or that are more agile at fleeing (i.e., smallest species), or that settle in the safest parts of the habitat (tree canopy; Götmark & Post, 1997). The latter finding agrees with the studies of da Cunha et al. (2017) and Dutour et al. (2017), who observed that birds more engaged in mobbing behavior were more protected by tall vegetation.

Our finding that species more exposed to predation responded less to heterospecific alarms indicates that these responses may be limited by species-specific predation risk. The more a species was preyed upon, the less likely it was to be stimulated by heterospecifics, a behavior that contrasts with the direct behavioral responses triggered by predators, in which it is often the species suffering high predation rates that show more pronounced response when exposed to the cues of predators. For example, vulnerable birds display more intense feather loss during predator attacks, a reaction that might confuse the predator and make it loose its hold (Møller et al., 2006), and more vulnerable species display faster escape times (Díaz et al., 2013). In our study, the cue is provided by heterospecifics rather than by noticing the predator directly, and individuals of highly vulnerable species that eavesdrop and alarm call may increase their probability of suffering an attack by attracting a predator that they have not yet located precisely. Therefore, despite the fact that the response behavior could warn conspecifics of danger, and thus be advantageous, it may be too costly for the most vulnerable species.

Our findings are in contrast with the previous results of da Cunha et al. (2017) and Dutour et al. (2017), who found that mobbing birds had higher, not lower, predation risk in their specific habitats, where diurnal pygmy owls (*Glaucidium* spp.) are the main predators. Unlike in these studies, pygmy owls do not occur in our study region, and we used diverse types of alarms that included not only those intended to deter close predators and that are highly detectable, like mobbing calls (Curio, 1978), but also high-frequency ‘seet’ or ‘hawk’ calls. The latter indicate a flying raptor at the distance, a context that is not comparable to that of mobbing. Many of these sounds did trigger a response, and we found no physical sound structure that was tightly associated with eliciting a heterospecific response, suggesting that mobbing calls are not the sole contagious alarm calls of birds. The difference between our results and those of da Cunha et al. (2017) and Dutour et al. (2017) suggest that the tendency of vulnerable species to alarm call in response to a heterospecific alarm call, or refrain from doing so, is context dependent (e.g., type of predators, visibility or location of the predators).

### Social acoustic facilitation

Stimulation by heterospecific alarm calls was particularly high in *Regulus regulus*, *Cyanistes caeruleus*, *Erithacus rubecula* and *Periparus ater*. Responsive species such as these could play a fundamental role in social facilitation—that is, enhance the fitness of coexisting species by means of social signals even if the signaler does not benefit (as opposed to social mutualism in which both parties benefit; e.g., Spottiswoode et al., 2016). Social acoustic facilitation has already been postulated at an intraspecific level (Dutour et al., 2019; Szymkowiak, 2022, 2024) among heterospecifics for bird cohesion calls during migration (Gayk et al., 2021) and songs during breeding (Rossetto & Laiolo, 2024). Moreover, social facilitation has been suggested to occur through learning from heteropecifics (Magrath et al., 2015b; Szymkowiak, 2021). However, our results do not seem to support the idea that the response is stronger after more familiar playbacks, likely because no training with novel sounds was conducted, and the playbacks used were sounds that the species already encountered, irrespective of the frequency of encounter. This, however, does not exclude the possibility that learning could have been a process that led the species to respond.

Further studies should focus on the functional roles of species that we identified as most responsive, which might be analogous to the role of attendant or core species described for mixed flocks (e.g., Dolby & Grubb, 1999; Mangini et al., 2023). A possible role of social facilitation by these species seems to be asymmetric, based only on being very responsive, but not on being more responded to, since we found no evidence that some species elicited more response than others. If at all, the highest percentage of response elicited was after the playback of *Turdus merula*, a species that was rarely stimulated to respond itself to heterospecific alarms. While future studies would benefit from larger sample sizes across species, our results support the interspecific differences in responsiveness and the existence of the acoustic stimulation after heterospecific alarm calls compared to silent control, a pattern that was not observed with acoustic suppression. Furthermore, future studies should also incorporate noise controls to ensure that responses are not triggered by any sound, thereby allowing a clearer interpretation of the observed behavior. However, species exhibited stronger responses to narrow-sense alarms, indicating that they did not respond indiscriminately to all types of stimuli. Although we cannot discard that these responses are driven by a general behavioral mechanism related to acoustic threats, the observed behavior would still have functional relevance. From an ecological perspective, it is plausible that less vulnerable species are more likely to respond to such signals, as they might perceive them as potential threats without incurring the same risks associated with responding, as more vulnerable species might.

## Conclusions

In forest habitats, where maintaining visual contact with predators is difficult, eavesdropping on heterospecifics may be a safe, and possibly efficient, anti-predator strategy (Gill & Bierema, 2013; Magrath et al., 2015b). This strategy helps birds to cope with predation in an environment that provides a lot of acoustic information and possibilities for acoustic interactions among co-occurring species.

## Supplementary Information

Below is the link to the electronic supplementary material.

Supplementary file1 (DOCX 3673 KB)

## Data Availability

Data used in this study are included as electronic supplementary material.
