# Supplementary material for: Eavesdropping and contagious alarming in bird communities
Source: Learn Behav. 2025 Jun 2;53(4):321–34. doi: 10.3758/s13420-025-00678-z (PMC12615574; doi:10.3758/s13420-025-00678-z)
Supplement: Supplementary file 1 — Supplementary file1 (DOCX 3673 KB) [file 13420_2025_678_MOESM1_ESM.docx]

**Supplementary Material**

**Supplementary tables**

**Table S1** Information on stimuli used as playback of alarm calls. XC: xenocanto (htpp://xenocanto.org) codes of the tracks broadcast during playback experiments. Track used to estimate the acoustic characteristics of the signal broadcast are depicted in bold. N: number of experiments conducted for each playback species. St: average percentage of vocal stimulation elicited in heterospecifics. Sup: average percentage of vocal suppression elicited. SPL: peak of sound pressure level of the tracks broadcasted

| **Playback species** | **XC** | **N** | **St** | **Sup** | **SPL (db)** |
| --- | --- | --- | --- | --- | --- |
| Certhia brachydactyla | **539340**; 405947 | 8 | 4.69 | 5.47 | 90 |
| Cyanistes caeruleus | 552947; **112754** | 9 | 12.50 | 2.78 | 90 |
| Erithacus rubecula | 436300; **92933;** 512416; 441920 | 7 | 6.25 | 4.46 | 84 |
| Fringilla coelebs | 131312; **527879;** 98872; 291970 | 7 | 9.82 | 8.04 | 88 |
| Lophophanes cristatus | **301897**; 348056 | 12 | 11.46 | 3.65 | 82 |
| Parus major | 170617; **96850;** 165496 | 9 | 6.94 | 8.33 | 87 |
| Periparus ater | **297792**; 149720 | 12 | 11.46 | 5.21 | 85 |
| Poecile palustris | **153824**; 698557 | 10 | 8.13 | 6.25 | 76 |
| Regulus ignicapilla | **148604**; 496349; 502419 | 8 | 6.25 | 3.13 | 72 |
| Sitta europaea | 118485; 154479; 462992;513541; **67240** | 8 | 6.25 | 3.13 | 88 |
| Sylvia atricapilla | 170715; 314032; **406323** | 7 | 5.36 | 2.68 | 71 |
| Troglodytes troglodytes | **401495**; 39895; 410464; 192659; 291636; 514082 | 8 | 5.47 | 0.00 | 74 |
| Turdus merula | 466533; **522349** | 9 | 14.58 | 3.47 | 78 |
| Turdus philomelos | 94685; 123586; 509456; 391414;199965; **112810** | 8 | 8.82 | 5.88 | 85 |

**Table S2** Literature sources in which narrow-sense alarms were recorded in the presence of predators for each playback species broadcast. [1]: Tietze, D. T., Martens, J., Sun, Y. H., & Päckert, M. (2008). Evolutionary history of treecreeper vocalisations (Aves: Certhia). Organisms Diversity & Evolution, 8(4), 305-324. [2]: Carlson, N. V., Healy, S. D., & Templeton, C. N. (2017). A comparative study of how British tits encode predator threat in their mobbing calls. *Animal Behaviour*, *125*, 77-92. [3]: Cramp S. (ed.), 1988 − Handbook of the birds of Europe, the Middle East, and North Africa: the birds of the Western Palearctic. [4]: Krams, I., & Krama, T. (2002). Interspecific reciprocity explains mobbing behaviour of the breeding chaffinches, *Fringilla coelebs*. Proceedings of the Royal Society of London. Series B: Biological Sciences, 269(1507), 2345-2350. [5] Naugler, C. T. (1993). Vocalizations of the Golden-Crowned Kinglet in Eastern North America. Journal of Field Ornithology, 346-351. [6] Walpole-Bond, J. O. H. N. (1931). Notes on the Songs and Cries of the British Nuthatch. British Birds, 25, 70-1. [7] Andrew, R. J. (1961). The Motivational Organisation Controlling the Mobbing Calls of the Blackbird (*Turdus merula*) Iv. a General Discussion of the Calls of the Blackbird and Certain Other Passerines. Behaviour, 18(3), 161-176. [8] Randler, C., & Vollmer, C. (2013). Asymmetries in commitment in an avian communication network. Naturwissenschaften, 100, 199-203. [9] Dutour, M., Léna, J. P., & Lengagne, T. (2017). Mobbing calls: a signal transcending species boundaries. Animal Behaviour, 131, 3-11. [10] East, M. (1981). Alarm calling and parental investment in the robin Erithacus rubecula. Ibis, 123(2), 223-230.; [11] Lack, D. (1954). CALL‐NOTES, ERITHACUS AND CONVERGENCE. Ibis, 96(2), 312-314. [12] Krama, T., Krams, R., Elferts, D., Sieving, K. E., & Krams, I. A. (2023). Selective selfishness in alarm calling behaviour by some members of wintering mixed-species groups of crested tits and willow tits. Philosophical Transactions of the Royal Society B, 378(1878), 20220102. [13] Krama, T., Krams, I., & Igaune, K. N. (2008). Effects of cover on loud trill‐call and soft seet‐call use in the crested tit Parus cristatus. Ethology, 114(7), 656-661.; [14] Carlson, N. V., Healy, S. D., & Templeton, C. N. (2020). What makes a ‘community informant’? [15] Grim, T. (2005). Host recognition of brood parasites: implications for methodology in studies of enemy recognition. The Auk, 122(2), 530-543.

| **Playback species** | **Literature sources of alarm repertoire** |
| --- | --- |
| *Certhia brachydactyla* | [1];[3] |
| *Cyanistes caeruleus* | [2]; [9] |
| *Erithacus rubecula* | [3]; [10]; [11] |
| *Fringilla coelebs* | [4]; [3]; [9]; [8] |
| *Lophophanes cristatus* | [2]; [12]; [13] |
| *Parus major* | [2]; [8]; [9] |
| *Periparus ater* | [2]; [9]; [14]; [9] |
| *Poecile palustris* | [2]; [8] |
| *Regulus ignicapilla* | [5]; [3] |
| *Sitta europaea* | [6]; [8] |
| *Sylvia atricapilla* | [3]; [15] |
| *Troglodytes troglodytes* | [3] |
| *Turdus merula* | [7]; [3] |
| *Turdus philomelos* | [3] |

**Table S3** Results of generalized least squared models analyzing the association between the % of vocal stimulation/suppression events elicited (dependent variable) and Sound Pressure Level SPL of the playback tracks broadcasted (fixed effect). Vocal stimulation (and suppression) events elicited were calculated as the percentage of experiments eliciting a response over the total of experiments conducted for each playback species (N = 14).

| **Suppression events elicited (%)** |  |  |  |  |
| --- | --- | --- | --- | --- |
|  | Value | Std.Error | t-value | p-value |
| *Sound pressure level SPL* | 0.16 | 0.08 | 1.94 | 0.08 |
| **Stimulation events elicited (%)** |  |  |  |  |
|  | Value | Std.Error | t-value | p-value |
| *Sound pressure level SPL* | 0.09 | 0.13 | 0.70 | 0.50 |

**Table S4** Literature sources of raw predation data from diet of the two raptors considered in this study. We selected percentages of all prey found in pellets and prey remains.

| ***Accipiter nisus*** | |
| --- | --- |
|  | Rodríguez, B., Rodríguez, A., Lorenzo, J. A., & Martínez, J. M. (2020). The Macaronesian Sparrowhawk diet in native and exotic forests. Ornis Fennica, 97, 00-00.  Gryz, J., & Krauze-Gryz, D. (2018). Density dynamics, diet composition and productivity of sparrowhawk Accipiter nisus L. population in central Poland. Forest Research Papers, 79(3), 245-251.  Zawadzka, D., & Zawadzki, J. (2001). Breeding populations and diets of the Sparrowhawk Accipiter nisus and the Hobby Falco subbuteo in the Wigry National Park (NE Poland). Acta ornithologica, 36(1), 25-31.  Petty, S. J., Patterson, I. J., Anderson, D. I. K., Little, B., & Davison, M. (1995). Numbers, breeding performance, and diet of the sparrowhawk Accipiter nisus and merlin Falco columbarius in relation to cone crops and seed-eating finches. Forest Ecology and Management, 79(1-2), 133-146.  Thiollay, J. M. (1967). Ecologie d’une population de rapaces diurnes en Lorraine. La Terre et la vie. |
| ***Accipiter gentilis*** | |
|  | Rebollo, S., García-Salgado, G., Pérez-Camacho, L., Martínez-Hesterkamp, S., Navarro, A., & Fernández-Pereira, J. M. (2017). Prey preferences and recent changes in diet of a breeding population of the Northern Goshawk Accipiter gentilis in Southwestern Europe. Bird Study, 64(4), 464-475.  Petronilho, J., & Vingada, J. V. (2002). First data on feeding ecology of Goshawk Accipiter gentilis during the breeding season in the natura 2000 site Dunas de Mira, Gândara e Gafanhas (Beira Litoral, Portugal). Airo.  Bijlsma, R. G. (2003). Trends en broedresultaten van roofvogels in Nederland in 2002. De takkeling, 11(1), 6-54.  Šotnár, K. (2000). Príspevok k hniezdnej biológii a potravnej ekológii jastraba velkého (Accipiter gentilis) na hornom Ponitrí. Buteo, 11, 43-50.  Toyne, E. P. (1998). Breeding season diet of the Goshawk Accipiter gentilis in Wales. Ibis, 140(4), 569-579.  Marquiss, M., & Newton, I. (1982). The goshawk in Britain. British Birds, 75, 243-260.  Bezzel, E., Rust, R., & Kechele, W. (1997). Nahrungswahl südbayerischer Habichte Accipiter gentilis während der Brutzeit. Orn. Anz, 36, 19-30.  Ivanovsky, V. V. (1998). Current status and breeding ecology of the Goshawk Accipiter gentilis in northern Belarus. Holarctic Birds of Prey, ADENEXWWGBP, Calamonte, Spain, 111-115.  PADIAL, J. M., BAREA, J. M., CONTRERAS, F. J., Avila, E., & PErez, J. (1998). Dieta del azor común (Accipiter gentilis) en las sierras béticas de Granada durante el periodo de reproducción. Ardeola, 45(1), 55-62.  Thiollay, J. M. (1967). Ecologie d’une population de rapaces diurnes en Lorraine. La Terre et la vie.  Rutz, C. (2004). Breeding season diet of Northern Goshawks Accipiter gentilis in the city of Hamburg, Germany. Corax, 19, 311-322.  Nielsen, J. T., & Drachmann, J. (1999). Prey selection of Goshawks Accipiter gentilis during the breeding season in Vendsyssel, Denmark. Dansk Orn Foren Tidsskr, 93, 85-90.  Zsolt, V. & Jozsef, R. (1993). Food and population dynamics of birds of prey. Aquila 100, 123–136.  Penteriani, V. (1997). Long-term study of a Goshawk breeding population on a Mediterranean mountain (Abruzzi Apennines, Central, Italy): density, breeding performance and diet. Journal of Raptor Research, 31, 308-312.  Opdam, P., Thissen, J., Verschuren, P., & Müskens, G. (1977). Feeding ecology of a population of GoshawkAccipiter gentilis. Journal für Ornithologie, 118(1), 35-51.  Verdejo, J. (1994). Datos sobre la reproducción y alimentación del azor (Accipiter gentilis) en un área mediterránea. Ardeola, 41, 37-43.  Kenward, R. E. (1979). Winter predation by goshawks in lowland Britain. British Birds, 72, 64-73. |

**Table S5** Pairwise matrix of phylogenetic distance among the study species. A multigene phylogeny was built by downloading 10,000 time-calibrated relaxed molecular clock trees from http://www.bird.tree.org [1] using the Hackett backbone of species [2]. Sequence data were available for all selected species. A maximum credibility clade tree was constructed with TreeAnnotator of BEAST 1.8.2 [3] in which we identified non-nested pairs of species. From the tree, we obtained a cophenetic distance matrix representing the pairwise phylogenetic distance between species with the function cophenetic in the R stats package (R Core Team). Species are indicated as follows: AegCau: *Aegithalos caudatus*; CerBra: *Certhia brachydactyla*; CyaCae: *Cyanistes caeruleus*; EriRub: *Erithacus rubecula*; FriCoe: *Fringilla coelebs*; ParMaj: *Parus major*; PerAte: *Periparus ater*; PyrUla: *Pyrrhula pyrrhula*; RegIgn: *Regulus ignicapilla*; SitEur: *Sitta europaea*; SylAtr: *Sylvia atricapilla*; TroTro: *Troglodytes troglodytes*; TurMer: *Turdus merula*; TurPhi: *Turdus philemelos*. [1]: Jetz, W., Thomas, G.H., Joy, J.B., Hartmann, K. & Mooers, A.O. (2012) The global diversity of birds in space and time. Nature, 491, 444–448 [2]: Hackett, S.J., Kimball, R.T., Reddy, S., Bowie, R.C., Braun, E.L., Braun, M.J., Chojnowski, J.L., Cox, W.A., Han, K.L., Harshman, J., Huddleston, C.J., Marks, B.D., Miglia, K.J., Moore, W.S., Sheldon, F.H., Steadman, D.W., Witt, C.C. & Yuri, T. (2008) A phylogenomic study of birds reveals their evolutionary history. Science, 320, 1763–1768. [3] Drummond, A.J., Nicholls, G.K., Rodrigo, A.G. & Solomon, W. (2002) Estimating mutation parameters, population history and genealogy simultaneously from temporally spaced sequence data. Genetics, 161, 1307–1320.

**Table S6** Pairwise matrix of trophic niche distance among the study obtained though *dist* function in R from a binary matrix of diet type and feeding substrate (insectivorous, granivorous, herbivorous, frugivorous, scavengers, rock, ground, grass, air, water, leaves, or bark), with data obtained from Laiolo et al. ([2018](https://link.springer.com/article/10.1007/s00265-023-03427-2#ref-CR37)). Species are indicated as follows: AegCau: *Aegithalos caudatus*; CerBra: *Certhia brachydactyla*; CyaCae: *Cyanistes caeruleus*; EriRub: *Erithacus rubecula*; FriCoe: *Fringilla coelebs*; ParMaj: *Parus major*; PerAte: *Periparus ater*; PyrUla: *Pyrrhula pyrrhula*; RegIgn: *Regulus ignicapilla*; SitEur: *Sitta europaea*; SylAtr: *Sylvia atricapilla*; TroTro: *Troglodytes troglodytes*; TurMer: *Turdus merula*; TurPhi: *Turdus philemelos*.

**Table S7** Average acoustic characteristics of the study species calls uttered to respond to heterospecific and conspecific playbacks and of the playback broadcasted more frequently. Acoustic characteristics are measured from power spectra (spectral parameters) and oscillograms (duration). Fpeak: peak frequency (logHz; frequency in the song with the greatest amplitude); Fbw: frequency bandwidth (logHz; measured as the difference between log transformed average maximum frequency and log transformed average minimum frequency; N: number of alarm calls measured for each species. *Turdus merula* never employed calls to respond to conspecific playback and *Garrulus glandarius* call was not broadcasted.

| **Calls to heterospecifics:** |  |  |  |  |
| --- | --- | --- | --- | --- |
|  | **Fpeak (log_10_Hz)** | **fbw (log_10_Hz)** | **duration (log_10_sec)** | **N** |
| *Cyanistes caeruleus* | 3.614 | 0.305 | 0.019 | 6 |
| *Erithacus rubecula* | 3.880 | 0.259 | -1.265 | 6 |
| *Fringilla coelebs* | 3.607 | 0.123 | -0.759 | 3 |
| *Garrulus glandarius* | 3.414 | 0.571 | -0.399 | 8 |
| *Parus major* | 3.750 | 0.558 | -0.098 | 6 |
| *Periparus ater* | 3.708 | 0.148 | -0.562 | 8 |
| *Regulus ignicapilla* | 3.857 | 0.058 | -0.884 | 9 |
| *Sitta europaea* | 3.489 | 0.251 | -0.882 | 3 |
| *Troglodytes troglodytes* | 3.795 | 0.322 | -0.212 | 6 |
| *Turdus merula* | 3.645 | 0.221 | -1.198 | 3 |
|  |  |  |  |  |
| **Calls to conspecifics:** |  |  |  |  |
|  | **Fpeak (log_10_Hz)** | **fbw (log_10_Hz)** | **duration (log_10_sec)** | **N** |
| *Cyanistes caeruleus* | 3.592 | 0.306 | -0.036 | 6 |
| *Erithacus rubecula* | 3.870 | 0.195 | -0.858 | 6 |
| *Fringilla coelebs* | 3.631 | 0.114 | -0.863 | 3 |
| *Garrulus glandarius* | - | - | - | - |
| *Parus major* | 3.599 | 0.522 | -0.241 | 6 |
| *Periparus ater* | 3.754 | 0.119 | -0.583 | 8 |
| *Regulus ignicapilla* | 3.858 | 0.063 | -0.789 | 9 |
| *Sitta europaea* | 3.507 | 0.275 | -0.751 | 3 |
| *Troglodytes troglodytes* | 3.823 | 0.325 | -0.261 | 6 |
| *Turdus merula* | - | - | - | - |
|  |  |  |  |  |
| **Playback broadcasted:** |  |  |  |  |
|  | **Fpeak (log_10_Hz)** | **fbw (log_10_Hz)** | **duration (log_10_sec)** |  |
| *Certhia brachydactyla* | 3.712 | 0.182 | -0.499 |  |
| *Cyanistes caeruleus* | 3.654 | 0.809 | 0.006 |  |
| *Erithacus rubecula* | 3.910 | 0.104 | -0.338 |  |
| *Fringilla coelebs* | 3.631 | 0.260 | -0.496 |  |
| *Lophophanes cristatus* | 3.575 | 0.421 | -0.447 |  |
| *Parus major* | 3.888 | 0.910 | -0.122 |  |
| *Periparus ater* | 3.808 | 0.449 | -0.654 |  |
| *Poecile palustris* | 3.606 | 0.571 | -0.058 |  |
| *Regulus ignicapilla* | 3.869 | 0.078 | -0.729 |  |
| *Sitta europaea* | 3.314 | 0.647 | -0.633 |  |
| *Sylvia atricapilla* | 3.667 | 1.218 | -1.141 |  |
| *Troglodytes troglodytes* | 3.790 | 0.566 | -0.257 |  |
| *Turdus merula* | 3.786 | 0.574 | -0.541 |  |
| *Turdus philomelos* | 3.717 | 0.824 | -0.923 |  |

**Table S8** Average percentage of variation in acoustic behavior (vocal stimulation and vocal suppression) in playback experiments for each focal species after all heterospecific alarm calls (HC), after narrow-sense heterospecific alarm calls (HC_n), after broad-sense alarm calls (HC_b) and after silent controls (SC).

|  | **VOCAL STIMULATION** | | | | **VOCAL SUPPRESSION** | | | |
| --- | --- | --- | --- | --- | --- | --- | --- | --- |
| Focal species | HC (%) | SC(%) | HC_n(%) | HC_b(%) | HC(%) | SC(%) | HC_n(%) | HC_b(%) |
| *Aegithalos caudatus* | 2.10 | 0.00 | 0.85 | 2.14 | 3.49 | 2.78 | 2.94 | 2.50 |
| *Certhia brachydactyla* | 5.88 | 0.00 | 6.34 | 6.41 | 3.88 | 2.78 | 4.52 | 3.03 |
| *Cyanistes caeruleus* | 18.41 | 5.56 | 25.14 | 15.38 | 4.52 | 11.11 | 6.13 | 2.78 |
| *Erithacus rubecula* | 15.45 | 8.33 | 17.63 | 9.23 | 4.46 | 13.89 | 4.68 | 3.18 |
| *Fringilla coelebs* | 7.83 | 5.56 | 12.13 | 3.46 | 6.46 | 8.33 | 4.52 | 8.48 |
| *Garrulus glandarius* | 8.68 | 11.11 | 12.11 | 3.81 | 4.48 | 8.33 | 2.62 | 7.78 |
| *Lophophanes cristatus* | 3.05 | 0.00 | 3.33 | 2.56 | 0.85 | 0.00 | 0.00 | 1.82 |
| *Parus major* | 13.34 | 2.78 | 12.61 | 17.31 | 3.02 | 2.78 | 4.17 | 3.18 |
| *Peripatus ater* | 15.44 | 2.78 | 12.18 | 16.67 | 3.91 | 5.56 | 0.93 | 4.09 |
| *Phylloscopus collybita* | 1.81 | 0.00 | 2.56 | 1.43 | 1.62 | 8.33 | 0.70 | 2.08 |
| *Poecile palustris* | 0.64 | 0.00 | 0.85 | 0.00 | 2.84 | 0.00 | 0.70 | 5.30 |
| *Pyrrula pyrrula* | 3.80 | 5.56 | 5.24 | 1.43 | 8.79 | 2.78 | 8.27 | 10.28 |
| *Regulus ignicapilla* | 21.75 | 0.00 | 26.72 | 15.13 | 3.02 | 13.89 | 0.00 | 8.33 |
| *Sitta europaea* | 5.56 | 2.78 | 6.78 | 3.46 | 3.68 | 5.56 | 2.08 | 5.00 |
| *Sylvia atricapilla* | 1.95 | 0.00 | 2.08 | 1.54 | 0.00 | 0.00 | 0.00 | 0.00 |
| *Troglodytes troglodytes* | 13.51 | 8.33 | 12.49 | 15.13 | 11.59 | 13.89 | 12.68 | 9.09 |
| *Turdus merula* | 5.40 | 2.78 | 7.35 | 1.92 | 9.30 | 8.33 | 15.67 | 0.91 |

**Supplementary figures**


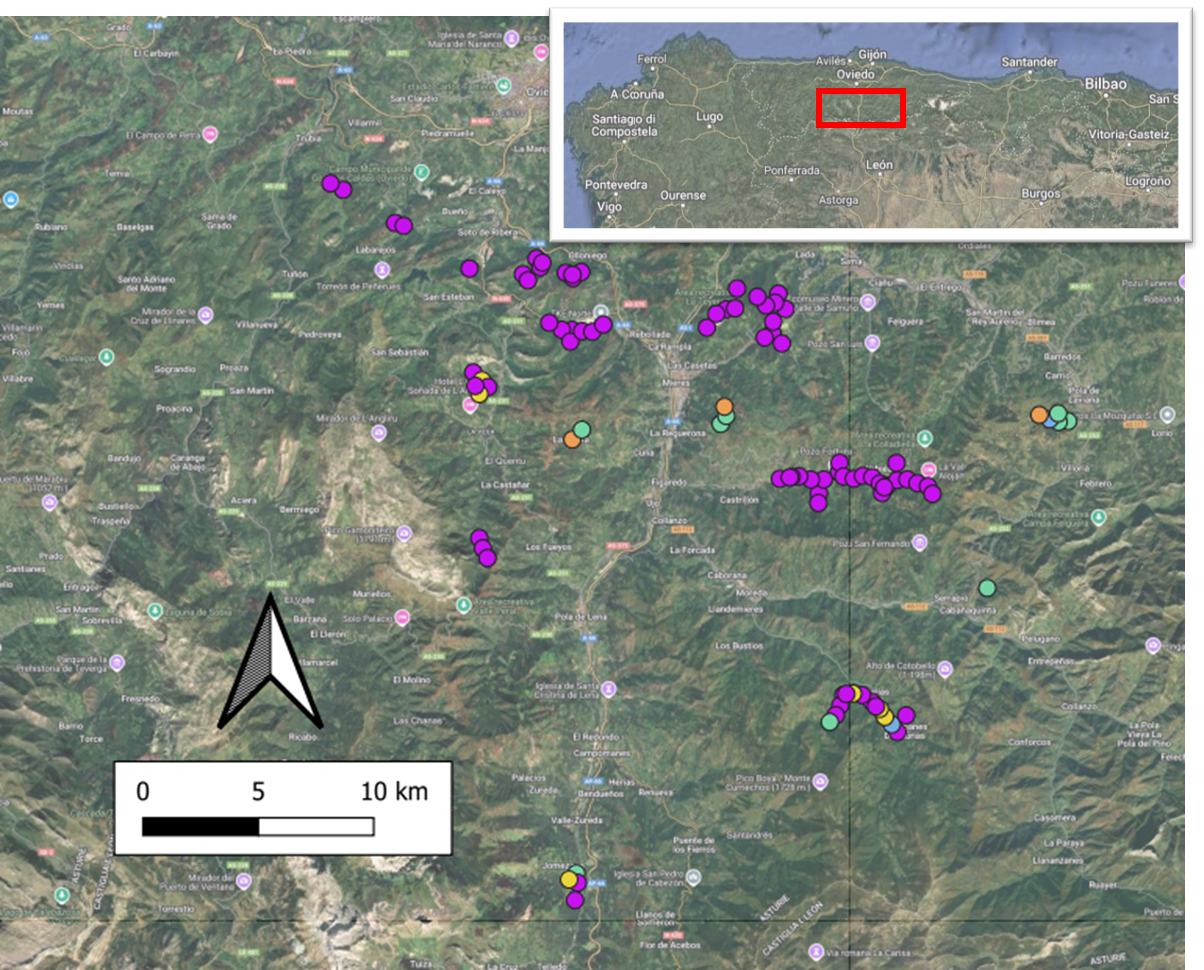


**Figure S1** Aerial photographs of the study area and locations of playback experiments. Playback trials were conducted in 84 localities. In 6 localities playback trials were conducted both in 2020 and 2022 broadcasting different species (yellow dots), in 2 localities playback trials were conducted in 2021 and 2022 (blue dots), in 3 localities trials were conducted only in 2021 (orange dots) in 9 localities trials were conducted only in 2022 (green dots) in and in the remaining localities playback trials were conducted only in 2020 (violet dots). The vast majority of the trials were conducted in 2020, with a total of 95 trials. In 2021 we only performed 5 trials, and 22 trials in 2022. The nearest locations of playback experiments were separated by 400 m, the farthest by 45 km. The median distance between locations was 15.29 Km. The figure was elaborated with QGIS and Google Hybrid as background map.

**
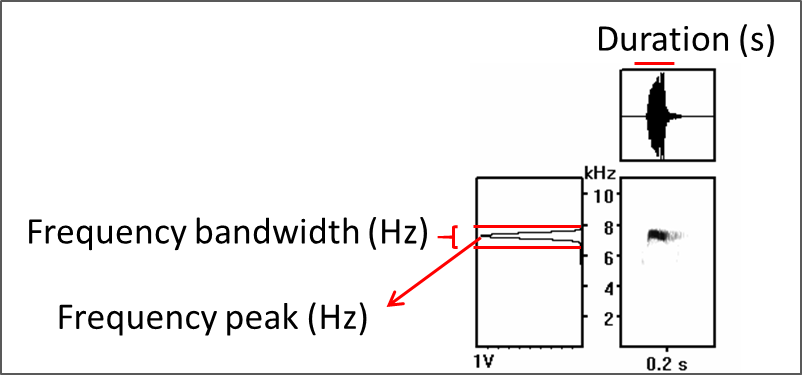
**

**Figure S2** Graphical representation of alarm call of a common firecrest (*Regulus ignicapilla*) from our study area, illustrating the acoustic measurements obtained with Avisoft SASLab Pro Software (Version 4.2) on the oscillogram (duration, top) and the mean power spectrum (frequency variables, left). The sounds measured were selected among the cleanest and loudest (high signal to noise ratio), thus maximum and minimum frequency were measured at the intersections between axes.


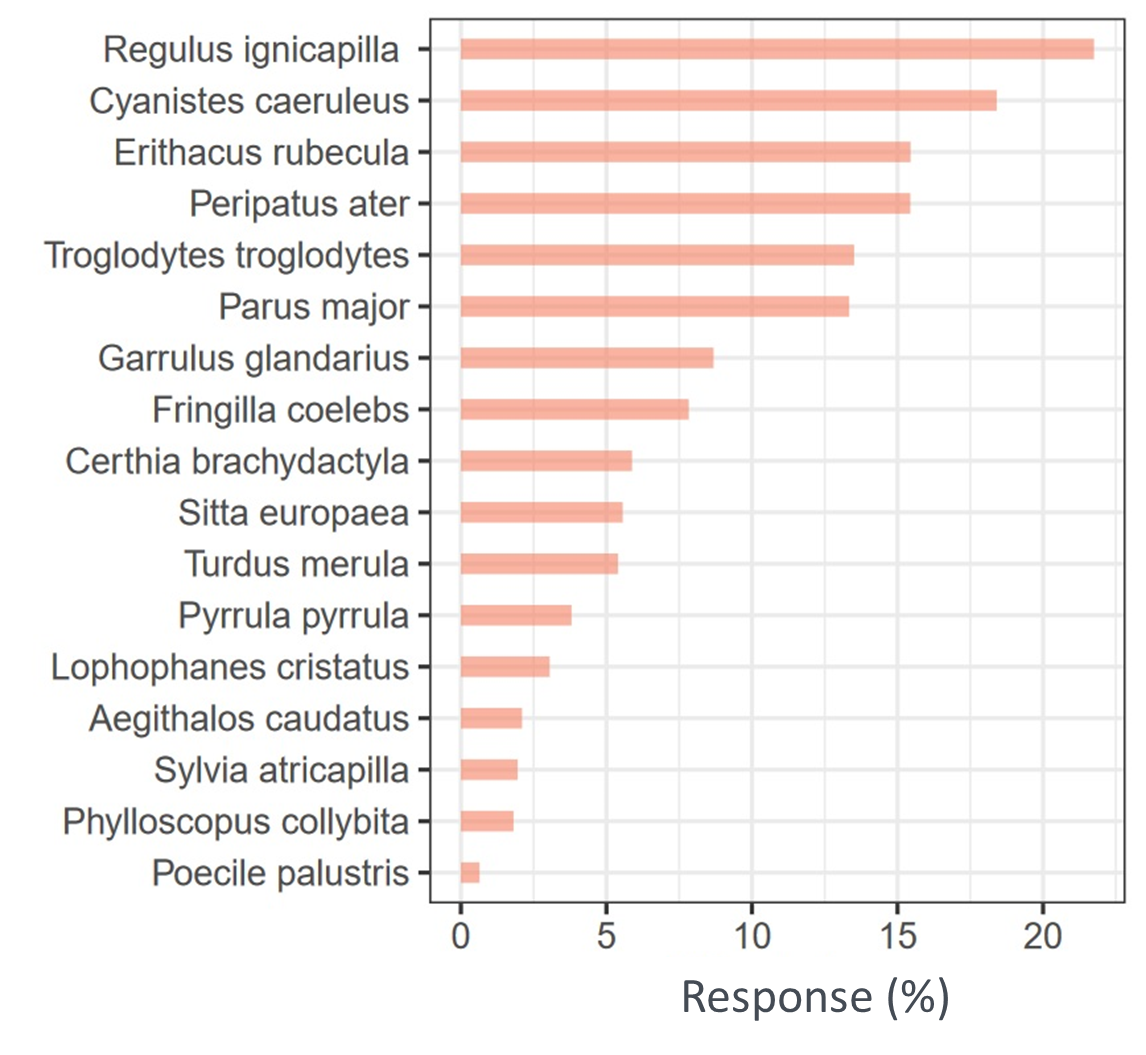


**Figure S3** Barplot representing the percentage of response (stimulation) to heterospecific playback experiments of the study species.
